# Intravenous thrombolysis and endovascular therapy for acute ischemic stroke in COVID-19: a systematic review and meta-analysis

**DOI:** 10.3389/fneur.2023.1239953

**Published:** 2023-08-15

**Authors:** Isabella Stuckart, Ahmed Kabsha, Timo Siepmann, Kristian Barlinn, Jessica Barlinn

**Affiliations:** ^1^Department of Neurology, Faculty of Medicine and University Hospital Carl Gustav Carus, Technische Universität Dresden, Dresden, Germany; ^2^Division of Health Care Sciences, Center for Clinical Research and Management Education Dresden, Dresden International University, Dresden, Germany

**Keywords:** acute ischemic stroke, COVID-19, reperfusion therapy, intravenous thrombolysis, endovascular therapy

## Abstract

**Background:**

The impact of COVID-19 on clinical outcomes in acute ischemic stroke patients receiving reperfusion therapy remains unclear. We therefore aimed to synthesize the available evidence to investigate the safety and short-term efficacy of reperfusion therapy in this patient population.

**Methods:**

We searched the electronic databases MEDLINE, Embase and Cochrane Library Reviews for randomized controlled trials and observational studies that investigated the use of intravenous thrombolysis, endovascular therapy, or a combination of both in acute ischemic stroke patients with laboratory-confirmed COVID-19, compared to controls. Our primary safety outcomes included any intracerebral hemorrhage (ICH), symptomatic ICH and all-cause in-hospital mortality. Short-term favorable functional outcomes were assessed at discharge and at 3 months. We calculated pooled risk ratios (RR) and 95% confidence intervals (CI) using DerSimonian and Laird random-effects model. Heterogeneity was evaluated using Cochran’s Q test and *I*^2^ statistics.

**Results:**

We included 11 studies with a total of 477 COVID-19 positive and 8,092 COVID-19 negative ischemic stroke patients who underwent reperfusion therapy. COVID-19 positive patients exhibited a significantly higher risk of experiencing any ICH (RR 1.54, 95% CI 1.16–2.05, *p* < 0.001), while the nominally increased risk of symptomatic ICH in these patients did not reach statistical significance (RR 2.04, 95% CI 0.97–4.31; *p* = 0.06). COVID-19 positive stroke patients also had a significantly higher in-hospital mortality compared to COVID-19 negative stroke patients (RR 2.78, 95% CI 2.15–3.59, *p* < 0.001). Moreover, COVID-19 positive stroke patients were less likely to achieve a favorable functional outcome at discharge (RR 0.66, 95% CI 0.51–0.86, *p* < 0.001) compared to COVID-19 negative patients, but this difference was not observed at 3-month follow-up (RR 0.64, 95% CI 0.14–2.91, *p* = 0.56).

**Conclusion:**

COVID-19 appears to have an adverse impact on acute ischemic stroke patients who undergo reperfusion therapy, leading to an elevated risk of any ICH, higher mortality and lower likelihood of favorable functional outcome.

**Systematic review registration:**

PROSPERO, identifier CRD42022309785.

## Introduction

Severe Acute Respiratory Syndrome Coronavirus 2 (SARS-CoV-2) caused a global pandemic of Coronavirus Disease 2019 (COVID-19) since its emergence in December 2019. By the time the World Health Organization declared the end to the COVID-19 global health emergency in May 2023, over 765 million confirmed cases of COVID-19 and more than 6.9 million reported deaths had been recorded worldwide (1). COVID-19 has been linked to a higher incidence of acute ischemic stroke, possibly due to disease-associated complications such as endothelial inflammation, hypercoagulopathy and cardiac thromboembolism (2–6).

Acute ischemic stroke is a leading cause of permanent disability in adults and death in the Western countries (7). COVID-19 patients with coincident acute ischemic stroke have been found to have worse outcomes, including higher mortality, compared to those without COVID-19 (6). Reperfusion therapies including intravenous thrombolysis (IVT) with tissue plasminogen activator (tPA) and endovascular therapy (EVT) are effective and approved treatments for mitigating the risk of long-term disability and death in acute ischemic stroke patients (8). However, COVID-19 patients are generally at a higher risk of systemic bleeding complications due to coagulation disorders, which might be exacerbated by these reperfusion therapies (9). Moreover, COVID-19 patients are frequently treated in designated COVID-19 units, which may not provide the same level of stroke care as stroke units, leading to suboptimal neurological and hemodynamic monitoring and potentially increasing the risk of early bleeding complications from reperfusion therapies (4).

As literature on the utilization of reperfusion therapies in COVID-19 positive ischemic stroke patients is limited, we conducted a systematic review and meta-analysis of available literature to explore the safety and short-term efficacy of reperfusion therapies in this patient population.

## Methods

### Protocol registration

The pre-specified protocol for this systematic review and meta-analysis was registered with the international prospective register of systematic reviews (PROSPERO; Registration No. CRD42022309785) and the methodology adhered to updated preferred reporting items for systematic reviews and meta-analyzes (PRISMA) guidelines (10). The PRISMA checklist is provided in the Supplementary material.

### Literature search and study eligibility

A systematic literature search using the electronic databases MEDLINE accessed by PubMed, Embase and Cochrane Library was performed from February 14 to March 8, 2022. The study eligibility criteria were defined in terms of: (1) *Participants*, which included acute ischemic stroke patients with concurrent laboratory-confirmed SARS-CoV-2 infection or COVID-19; (2) *Intervention*, involving reperfusion therapy by means of IVT, EVT or a combination of both; (3) *Controls*, consisting of a comparator group of acute ischemic stroke patients without SARS-CoV-2 infection or COVID-19 who were concurrently recruited; (4) *Outcomes*, which encompassed safety outcomes such as any intracerebral hemorrhage (ICH), symptomatic ICH, and all-cause in-hospital mortality, as well as short-term functional outcomes as indicated by the modified Rankin Scale (mRS) score at discharge or at 3 months; and (5) *Study design*, which included randomized controlled trials, observational cohort or case–control studies, or case series including at least 10 patients.

The search strategy was pre-defined without language restrictions and encompassed all publications from December 01, 2019 until our last search date March 8, 2022. Two reviewers (IS and AK) conducted the literature search independently and assessed all identified articles by screening of titles, abstracts and full texts using citation manager software to remove duplicates. In case of any discrepancies, a third investigator (JB) was consulted and disagreement was resolved by consensus. The search strings included various relevant terms and their combinations related to stroke and COVID-19 including “stroke,” “cerebrovascular disease,” “ischemic stroke,” “ischaemic stroke,” “brain ischemia,” “cerebral ischemia,” “embolic stroke,” “cerebrovascular disorders,” “coronavirus,” “COVID,” “COVID-19,” “2019-nCoV,” “severe acute respiratory syndrome coronavirus 2,” and “SARS-CoV-2.” No additional limits or filters were applied. The complete search algorithm is provided in the Supplementary materials. A snowball search in bibliographies of identified full-text articles and relevant review articles was also performed. If aforementioned outcomes of interest were not reported in eligible studies, the corresponding authors were asked to provide these data. Failure to provide at least one outcome of interest resulted in study exclusion. Furthermore, studies that did not confirm SARS-CoV-2 or COVID-19 cases by laboratory criteria (i.e., positive rapid antigen/PCR test) were considered unsuitable and excluded.

### Data collection and data items

The extracted information from full text articles included first authors names, publication year, study design, sample size, total number of acute ischemic stroke patients with SARS-CoV-2 or COVID-19, absolute numbers of acute ischemic stroke patients without SARS-CoV-2 or COVID-19, patients’ demographics including age and sex, baseline stroke severity using the National Institutes of Health Stroke Scale (NIHSS) score, reperfusion therapy time metrics, and the absolute numbers of aforementioned outcome events. All data were independently collected by two reviewers (IS and AK) and inserted into a standardized data extraction form (Excel; Microsoft, Redmond, WA, United States).

### Risk of bias assessment

We employed the Risk Of Bias In Non-randomized Studies - of Exposures (ROBINS-E) tool for quality control and bias assessment of included studies (11). Details of bias assessment for each study are listed in the Supplementary materials.

### Statistical analysis

Categorical variables were reported as percentages, while continuous variables were presented as either mean ± standard deviation (SD) or median and interquartile range (IQR). The modified Wald method was used for computation of corresponding 95% confidence intervals (95%CI). To determine the pooled relative risks (RR) and their 95%CI for each categorial outcome of interest, we used a DerSimonian and Laird random-effects model (12). In addition, weighted mean differences (WMD) were calculated for continuous data such as time metrics. In studies where only the IQR was provided, we estimated the SD by dividing the range by 1.35 (13). Continuity correction of 0.5 was applied to studies with a zero cell. Sensitivity analyzes were performed on studies with consistent definitions for corresponding outcomes. We assessed the heterogeneity across the included studies using Cochran Q and the Higgins *I^2^* test. Specifically, *I^2^* values of 0 to 40% indicated absent or low heterogeneity, 30 to 60% indicated moderate heterogeneity, 50 to 90% indicated substantial heterogeneity, and 75 to 100% indicated considerable heterogeneity (14). Significance level of heterogeneity was set at *p* < 0.1. To examine the possibility of publication bias, we utilized Egger’s test. We also visually inspected the corresponding funnel plots for the presence of small study effects. All statistical analyzes were conducted using STATA (version 16, StataCorp, College Station, TX). Statistical significance was set at *p* < 0.05.

## Results

### Systematic literature review

**Figure 1:**
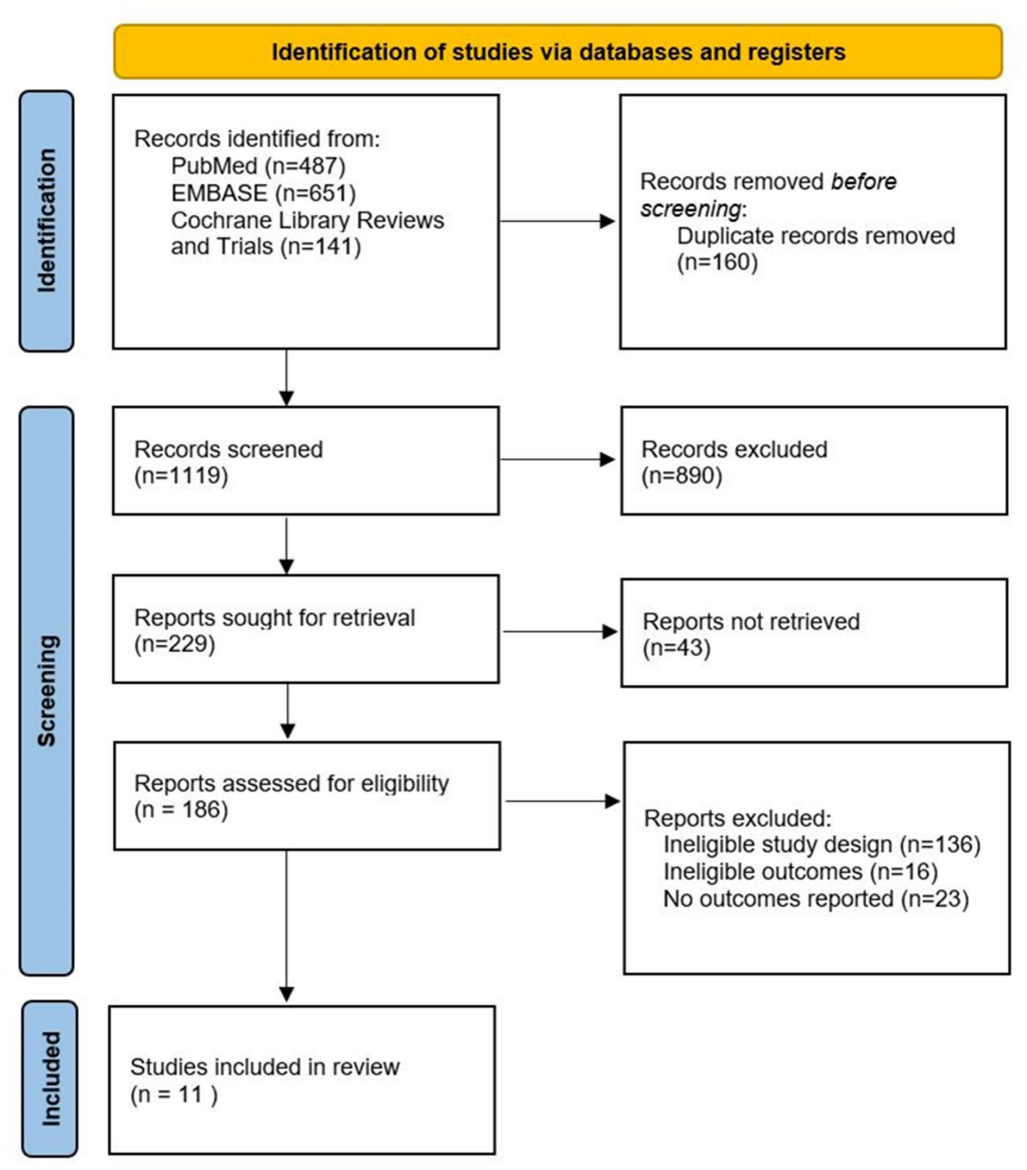
Flow chart of study selection.

Out of 1,279 titles retrieved from the electronic databases and the bibliographies from published articles, 160 were excluded due to duplication. After screening 1,119 abstracts, 186 full articles were evaluated for eligibility. Ultimately, 11 studies with a total of 8,569 acute ischemic stroke patients with (*n* = 477) and without (*n* = 8,092) concurrent SARS-CoV-2 infection or COVID-19 were included for quantitative synthesis (15–25). Two corresponding authors provided necessary outcome data upon request (24, 25). The systematic screening and selection process is shown in Figure 1. Of the included studies, eight were retrospective observational studies and three were prospective. The majority of the included studies (*n* = 7) provided data on a combination of IVT and EVT (15, 19–22, 24, 25), while three studies provided data on EVT (16–18). One study focused on IVT only (23). Table 1 summarizes the characteristics of the included studies.

**Table 1:** Characteristics of the studies included in the quantitative data synthesis.

Study	Study design	Study size, n*		Age, years, mean ± SD/median (IQR)		Female, *n* (%)		NIHSS, median (IQR)		Intravenous thrombolysis, *n* (%)		Endovascular therapy, *n* (%)		Door-to-needle time, min, median (IQR)		Door-to-groin-time, min, median (IQR)	
		COVID-19	Non-COVID-19	COVID-19	Non-COVID-19	COVID-19	Non-COVID-19	COVID-19	Non-COVID-19	COVID-19	Non-COVID-19	COVID-19	Non-COVID-19	COVID-19	Non-COVID-19	COVID-19	Non-COVID-19
Al Kasab et al. (2020) (15)	Prosepective	13	445	58 (50–71)	72 (60–80)	5 (38.5)	205 (46.1)	19 (16–24)	15 (10–20)	4 (30.8)	180 (40.4)	13 (100)	445 (100)	NA	NA	56 (37–150)	82 (50–127)
Asteggiano et al. (2021) (16)	Retrospective	5	28	66.1 ± 10.8	75.4 ± 8.8	0	18 (64.3)	NA	NA	NA	NA	5 (100)	28 (100)	NA	NA	NA	NA
De Havenon et al. (2020) (17)	Retrospective	104	3,061	NA	NA	33 (31.7)	1,490 (48.7)	NA	NA	NA	NA	104 (100)	3,061 (100)	NA	NA	NA	NA
Gabet et al. (2021) (18)	Retrospective	55	2036	66.9	70.9	19 (34)	1,059 (52)	NA	NA	NA	NA	55 (100)	2036 (100)	NA	NA	NA	NA
Qureshi et al. (2021) (19)	Retrospective	96	1,588	NA	NA	33 (34.4)	783 (49.3)	NA	NA	55 (57.3)	751 (47.3)	35 (36.5)	729 (45.9)	NA	NA	NA	NA
Sasanejad et al. (2021) (20)	Prosepective	101	444	68.19 ± 13.3	68.34 ± 14.5	41 (40.6)	200 (45.2)	13 (9–19)	11 (7–17)	101 (100)	444 (100)	11 (10.9)	55 (12.4)	41 (24.5–60)	40 (25–58)	NA	NA
Pezzini et al. (2021) (21)	Retrospective	34	262	76 (63–82.25)	74 (61–80)	10 (29.4)	132 (50.4)	12 (7–20.25)	10 (6–16)	16 (47.1)	99 (37.8)	18 (52.9)	163 (62.2)	215 (184–258.75)^#^	185 (145–225)^#^	245 (207.5–294)^‡^	194.5 (150–255)^‡^
Requena et al. (2020) (22)	Retrospective	10	19	70.8 ± 14.8	71.0 ± 15.9	4 (40)	8 (42.1)	18 (11–25)	17 (9–21)	1 (10)	5 (26.3)	10 (100)	19 (100)	NA	NA	118 (45.5–134.5)	75 (46–93.5)
Sobolewski et al. (2021) (23)	Retrospective	22	48	74.5 ± 7.9	72.9 ± 12.8	7 (34.5)	27 (58)	11 (3–20)	6.5 (2–25)	22 (100)	48 (100)	0	0	52 (15–123)	61 (10–170)	NA	NA
Fuentes et al. (2021) (24)	Retrospective	30	138	NA	NA	NA	NA	NA	NA	25 (83.3)	81 (58.6)	17 (56.6)	90 (65.2)	61^$^	48.2^$^	232.2^$^	242^$^
Genchi et al. (2022) (25)	Prosepective	7	23	70.9 ± 12.4	74.7 ± 9.6	3 (42.9)	13 (56.5)	24 (20–26)	16 (9–22)	2 (28.6)	7 (30.4)	7 (100)	23 (100)	NA	NA	330 (255–495)^‡^	280 (193–675)^‡^

NA indicates not available; SD, standard deviation; IQR, interquartile range; NIHSS, National Institutes of Health Stroke Scale Score.

*Numbers of patients correspond to available outcome data in the included studies (and do not account for missing data); ^#^onset-to-needle time; ^$^number corresponds to mean, standard deviation not available; ^‡^onset-to-groin time.

Among the COVID-19 positive stroke patients included in the studies, 226 patients (47.4%; 95%CI, 42.9–51.9) received IVT, 275 patients (57.7%; 95%CI, 53.2–62) underwent EVT, and a total of 44 patients (9.2%; 95%CI, 6.9–12.2) received both treatments.

### Quantitative analysis of safety

Among the included studies, six provided data on any ICH (15, 19–23), while five studies reported on symptomatic ICH (15, 20–23). The definition of any ICH relied on radiological evidence of intracerebral blood, while symptomatic ICH mostly required a neurological deterioration of at least 4 points in the NIHSS score based on the Heidelberg bleeding classification (15, 20, 21, 23, 26). One study did not provide details on symptomatic ICH definition (22). The overall rate of any ICH was 17% (95%CI, 13–21.9) in the COVID-19 positive group and 10.6% (95%CI, 9.5–11.8) in the control group. The overall rate of symptomatic ICH was 3.9% (95%CI, 1.8–7.9) in the COVID-19 positive group and 3.9% (95%CI, 2.9–5.1) in the control group.

COVID-19 positive stroke patients were found to have a significantly higher risk of developing any ICH following reperfusion therapy compared to COVID-19 negative patients (RR 1.54, 95%CI, 1.16–2.05; *p* < 0.001), with no evidence of heterogeneity between the studies (*I*^2^ = 0%, *p* = 0.65). Although the risk of symptomatic ICH appeared to be nominally increased in COVID-19 positive acute ischemic stroke patients compared to COVID-19 negative patients (RR 2.04, 95%CI, 0.97–4.31), this association did not reach statistical significance (*p* = 0.06). There was no evidence of heterogeneity between the studies (*I*^2^ = 0%, *p* = 0.76).

**Figure 2:**
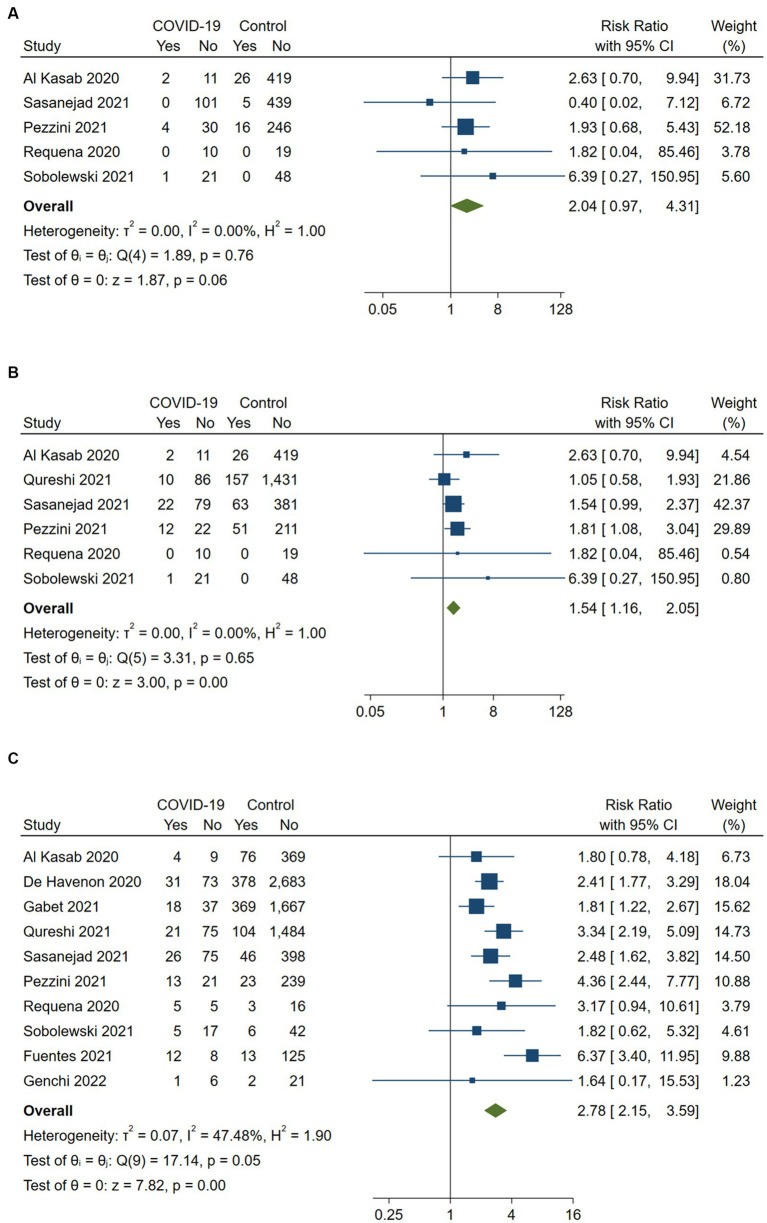
Pairwise meta-analysis of all available studies on **(A)** symptomatic intracerebral hemorrhage, **(B)** any intracerebral hemorrhage and **(C)** in-hospital mortality between COVID-positive and COVID-negative stroke patients receiving intravenous or endovascular therapy.

According to 10 included studies, in-hospital mortality for COVID-19 positive stroke patients who received any reperfusion therapy was 28.8% (95%CI, 24.9–33.1) in the treatment group and 12.7% (95%CI, 11.9–13.4) in the control group (15, 17–25). COVID-19 positive stroke patients who received any reperfusion therapy had significantly higher in-hospital mortality compared to COVID-19 negative stroke patients (RR 2.78, 95%CI 2.15–3.59, *p* < 0.001). There was moderate heterogeneity across the included studies (*I*^2^ = 47.5%, *p* = 0.05). The corresponding forest plots are depicted in Figure 2.

### Quantitative analysis of short-term efficacy

**Figure 3:**
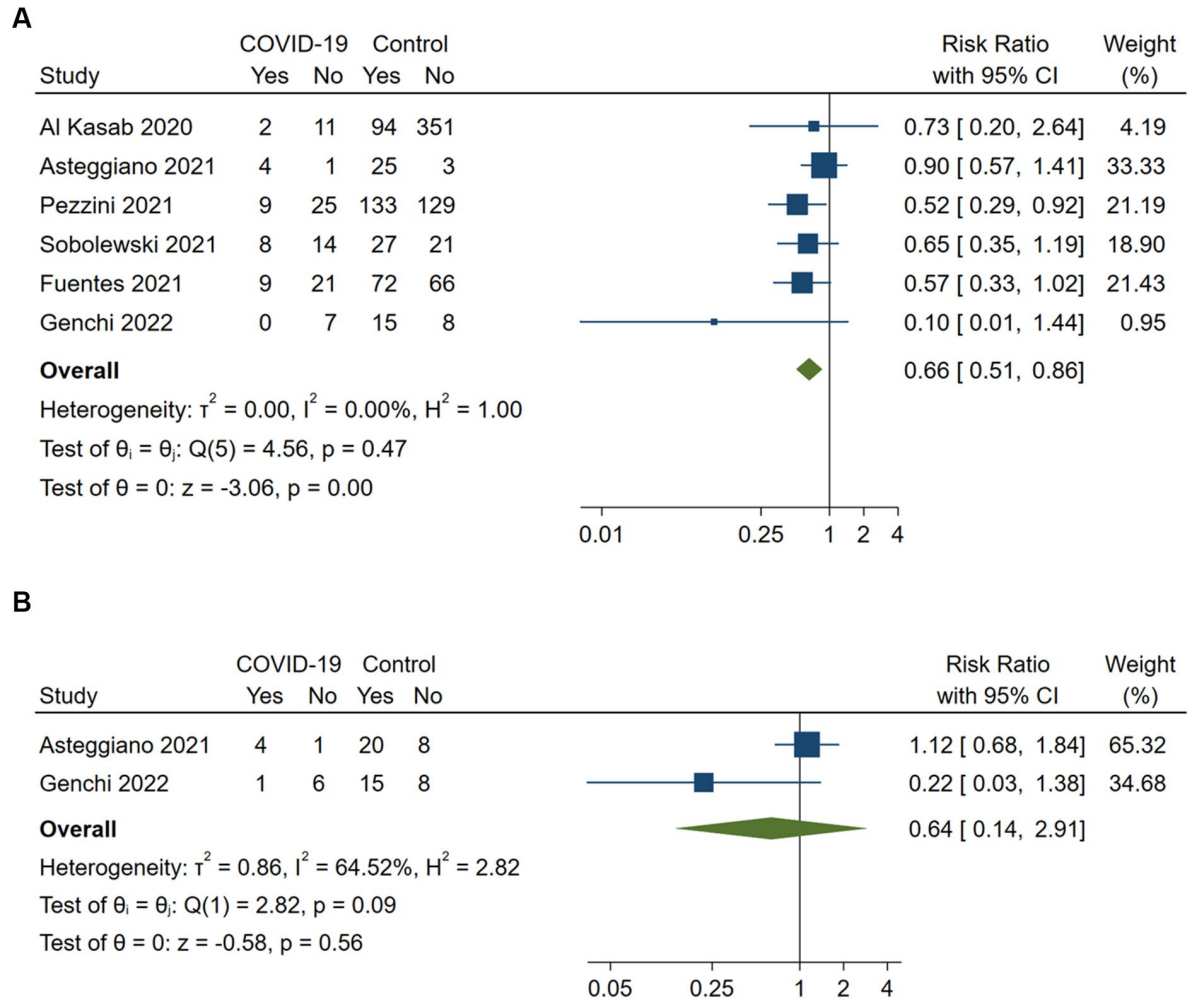
Pairwise meta-analysis of all available studies on favorable functional outcome **(A)** at discharge and **(B)** at 3 months between COVID-positive and COVID-negative stroke patients receiving intravenous or endovascular therapy.

Six studies assessed favorable functional outcomes at discharge defined as an mRS of 0 to 2 (*n* = 5) or mRS of 0 to 1 (*n* = 1) (15, 16, 21, 23–25). COVID-19 positive stroke patients had a significantly lower likelihood of achieving a favorable functional outcome at discharge compared to COVID-19 negative patients (RR 0.66, 95%CI 0.51–0.86; *p* < 0.001). No heterogeneity was observed between the studies (*I*^2^ = 0%, *p* = 0.47; Figure 3). A sensitivity analysis including the five studies with an mRS of 0 to 2 as the favorable outcome definition confirmed the robustness of the results (RR 0.57, 95%CI 0.41–0.79; *p* < 0.001), with no heterogeneity (*I*^2^ = 0%, *p* = 0.73).

Two studies reported on favorable functional outcomes (mRS 0 to 1) at 3 months (16, 25). The data synthesis suggested that COVID-19 positive stroke patients had a lower likelihood of achieving a favorable functional outcome at 3 months compared to COVID-19 negative patients (RR 0.64, 95%CI 0.14–2.91; *p* = 0.56). However, these results did not reach statistical significance and showed substantial heterogeneity (*I*^2^ = 64.5%, *p* = 0.09).

### Quantitative analysis of time metrics

**Figure 4:**
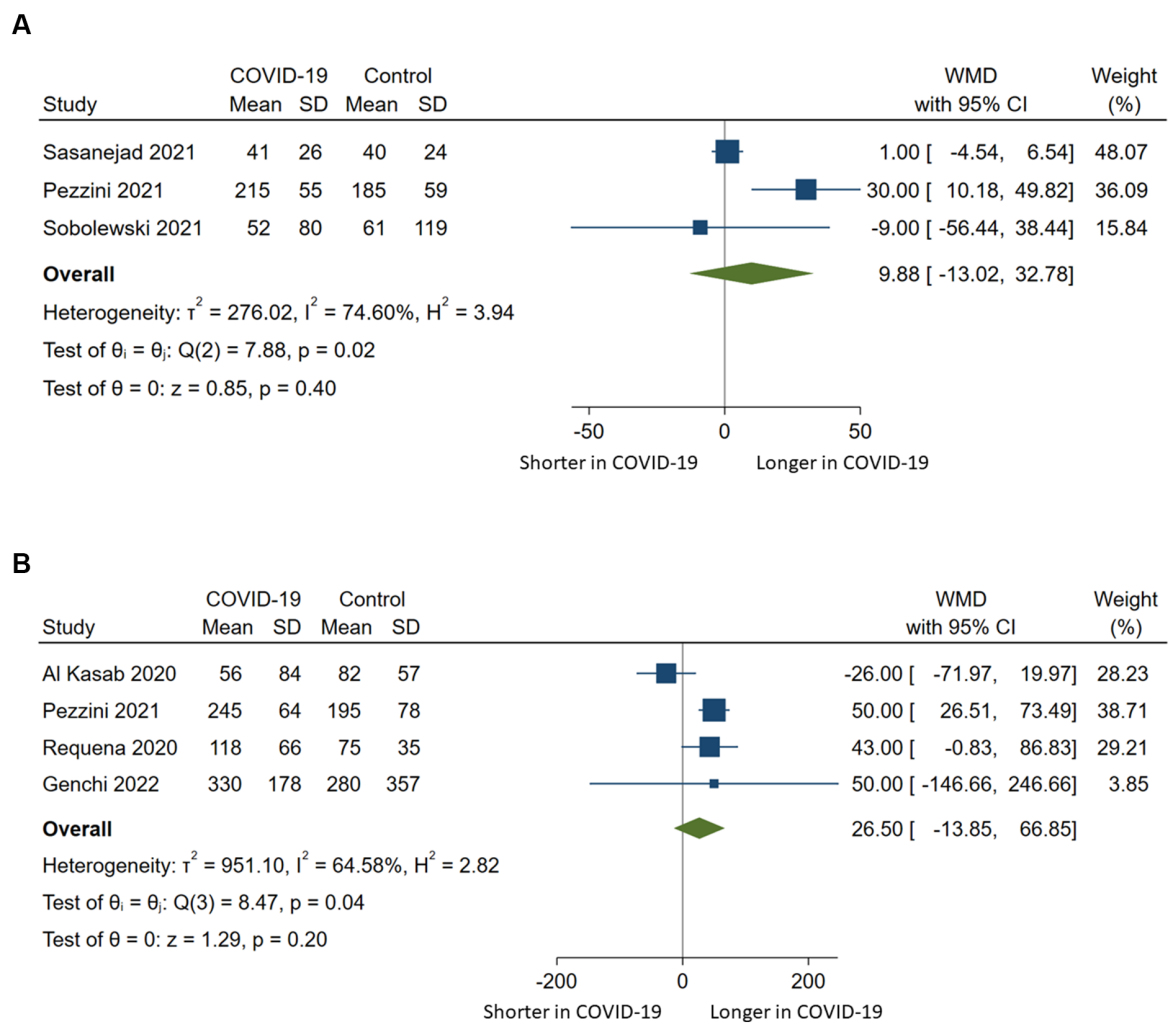
Pairwise meta-analysis comparing **(A)** door-to-needle times and **(B)** onset-to-groin or door-to-groin times among stroke patients with COVID-19 compared to those without COVID-19 who underwent intravenous or endovascular therapy. WMD indicates weighted mean difference.

Door-to-needle times were reported in three studies (20, 21, 23) and door-to-groin or onset-to-groin times in four studies (15, 21, 22, 25). The synthesis of the available data did not show a significant difference in terms of door-to-needle time (WMD 9.88, 95%CI −13.02-32.78, *p* = 0.4) or onset-to-groin or door-to-groin time (WMD 26.5, 95%CI −13.85-66.85, *p* = 0.2) between COVID-19 positive and controls. There was substantial heterogeneity observed across the included studies (Figure 4).

### Additional analysis

Synthesis of data provided by three studies on the necessity of mechanical ventilation revealed that stroke patients who tested positive for COVID-19 had a significantly higher risk of ventilation dependency compared to ischemic stroke patients without COVID-19 (RR 1.58, 95%CI 1.03–2.44, *p* = 0.037) (15, 17, 19). However, this association exhibited substantial heterogeneity (*I*^2^ = 73.7%, *p* = 0.02).

### Bias and quality control assessment

Publication bias assessment was only conducted for in-hospital mortality due to a limited number of studies meeting the eligibility criteria for other outcome variables. This decision aligns with the Cochrane Handbook for Systematic Reviews of Interventions, which recommends a minimum of 10 studies for appropriate bias assessment (27). The findings indicated no presence of publication bias, supported by a value of *p* >0.05 for Egger’s test and the absence of funnel plot asymmetry. Supplementary materials also contain the results of the ROBINS-E quality control assessment for the 11 included studies.

## Discussion

This systematic review and meta-analysis highlights a higher risk of any ICH and in-hospital death following reperfusion therapy in acute ischemic stroke patients with COVID-19 compared to those without COVID-19. However, there was no significant increase in the risk of symptomatic ICH among COVID-19 positive stroke patients. These findings might indicate that the increased mortality in these patients is likely attributable to COVID-19 and its associated complications rather than bleeding complications arising from reperfusion therapies.

Intracerebral hemorrhage is a significant complication that can occur after acute reperfusion therapy in acute ischemic stroke (26, 28). Current understanding indicates that ICH primarily results from tPA-related coagulopathy, blood–brain barrier disruption and hyperperfusion injury (29, 30). Nevertheless, hemorrhagic transformation of infarcted brain tissue, frequently detected through routine neuroimaging following reperfusion therapy, does not always have a negative impact on clinical outcomes (26, 30). Clinical significance becomes apparent when larger hematomas occur within the infarcted brain tissue and are accompanied by neurological deterioration, as indicated by an increase in the NIHSS score. Symptomatic intracerebral hemorrhages are strongly associated with unfavorable functional outcomes and increased mortality (28, 29). In our meta-analysis, COVID-19 positive patients with acute ischemic stroke displayed a heightened risk of any ICH following reperfusion therapy. Although the risk of symptomatic ICH was nearly doubled compared to COVID-19 negative patients, this association did not reach statistical significance and thus should be cautiously interpreted as potential indication of harm. In a recent multicenter study involving 853 COVID-19 positive ischemic stroke patients who received intravenous thrombolysis and/or EVT, a statistically significant 1.5-fold increased rate of sICH was observed compared to non-COVID-19 controls (9). This finding, which aligns with our analysis showing a nominally increased risk of sICH, could be attributed to the larger sample size utilized in the multicenter study compared to our data synthesis. Several factors have been identified as contributing to bleeding complications in ischemic stroke patients undergoing reperfusion therapy, including higher age, higher baseline NIHSS scores, elevated glucose levels, low platelet count and increased thrombin time at admission as well as inadequate blood pressure control (28, 29). Abstracted data from the included studies suggest that COVID-19 positive ischemic stroke patients had more severe strokes than COVID-19 negative patients (15, 20–23, 25). The risk of ICH in COVID-19 positive patients might be further enhanced due to pathophysiological mechanisms associated with COVID-19 including dysfunction of the renin-angiotensin system leading to reduced ACE2 expression, hypertension, elevated D-dimer and tPA plasma levels, as well as cerebral endothelial dysfunction caused by inflammatory factors (25, 31).

Our data synthesis indicates that COVID-19 can lead to unfavorable outcomes in patients with acute ischemic stroke. COVID-19 is associated with common complications such as severe pneumonia, respiratory failure, kidney and hepatic dysfunction, dysregulated inflammatory response resulting in septic shock, and cardiac arrest (32). These complications may significantly contribute to poor outcomes following reperfusion therapy. Previous studies have consistently identified COVID-19 as a significant predictor of mortality in acute ischemic stroke patients, regardless of the treatment method (33). Moreover, in COVID-19 positive ischemic stroke patients who underwent reperfusion therapy, there is a higher likelihood of experiencing acute respiratory and kidney failure, septic shock, cardiac arrest, and requiring mechanical ventilation compared to COVID-19 negative patients (19, 34). Consistently, our pooled analysis of three studies showed an increased risk of ventilation dependency in COVID-19 positive ischemic stroke patients compared to COVID-19 negative patients. It is worth noting that COVID-19 positive stroke patients often have more severe strokes, as indicated by higher baseline NIHSS scores, which may have contributed to worse functional outcomes in this pooled patient population (35, 36). Lastly, cardiovascular risk factors commonly observed in patients with ischemic stroke have been shown to be associated with a higher risk of severe COVID-19 (37). This association may further diminish the chances of favorable outcomes in patients with ischemic stroke and COVID-19.

Based on the available data from the studies included in our meta-analysis, it appears that COVID-19 did not lead to a significant delay in starting reperfusion therapy. However, there was a notable finding in one study where a significant delay was observed in both door-to-needle and door-to-groin time for COVID-19 positive stroke patients when COVID-19 was suspected and confirmed with chest CT (24). These findings are surprising because one might expect treatment delays due to pre-clinical hygienic measures, prolonged intra-hospital processes through preventive measures, and swabbing for COVID-19 testing, which could potentially result in a missed therapeutic window, fewer implemented reperfusion therapies, and worse outcomes, as suggested by some studies (4, 19, 33, 35). Therefore, it is possible that the worse outcomes observed in COVID-19 positive ischemic stroke patients could be associated with pathophysiological aspects of COVID-19 disease itself rather than delays in reperfusion therapy.

Our meta-analysis demonstrates several strengths including an comprehensive literature review involving two independent reviewers, strict adherence to standardized methodological criteria guided by the PRISMA statement and the ROBINS-E tool for risk of bias assessment, and prior registration with PROSPERO. A significant contribution of our study is the inclusion of four studies that were not encompassed in a recently published meta-analysis on the same topic (38). This inclusion expands the existing body of knowledge regarding the safety and short-term efficacy of reperfusion therapies in acute ischemic stroke patients with COVID-19. Importantly, we implemented rigorous measures to prevent potential overlap in patient samples within our analysis, addressing limitations identified in the previous meta-analysis where an overlap between two studies was observed (38–40). Furthermore, we excluded historical controls from our study considering that stroke outcomes during the pandemic could have been influenced by various factors, not only COVID-19 but also in-hospital cohorting and isolation strategies (4). Despite the differences in study selection, it is noteworthy that both meta-analyzes yielded comparable outcomes, underscoring the robustness of our findings.

Nevertheless, it is essential to acknowledge the limitations of our study. Firstly, the small sample size, particularly among ischemic stroke patients with COVID-19, restricts the generalizability of our findings to a broader population. Secondly, the absence of patient-level data on common confounding variables, such as age, baseline stroke severity, and the presence and location of large vessel occlusion, prevented us from performing meta-regression analysis. Additionally, the unavailability of patient-level data on COVID-19 severity hindered our ability to differentiate between unfavorable outcomes caused by the disease itself or complications arising from reperfusion therapy. Thirdly, due to possible impairment of consciousness in patients with severe COVID-19, it remains unclear how many patients eventually experienced asymptomatic or symptomatic ICH. Consequently, the actual symptomatic ICH rate could potentially be higher than reported. Fourthly, it is important to note that the majority of studies included in our meta-analysis recruited patients within the first year of the pandemic, aligning with the emergence of various SARS-CoV-2 variants, increasing vaccination rates and the availability of specific COVID-19 treatments. Our findings therefore may be prone to time-varying bias and may not necessarily be generalizable to current stroke patients with COVID-19. Lastly, the possibility of selection bias cannot be ignored, as patients may have been eligible for reperfusion therapy only if their treating physicians deemed the risk of bleeding complications to be low.

## Conclusion

Our meta-analysis indicates that acute ischemic stroke patients tested positive for COVID-19 and undergo reperfusion therapy might be at higher risk of unfavorable outcomes compared to stroke patients without COVID-19. To optimize treatment strategies for COVID-19 positive stroke patients, further studies are necessary to explore the underlying mechanisms that contribute to these potential worse outcomes.

## Data availability statement

The raw data supporting the conclusions of this article will be made available by the authors, without undue reservation.

## Author contributions

IS, AK, and JB: conceptualization of study design and supervision. IS, AK, JB, and KB: data acquisition, analysis and interpretation, manuscript writing and revision. TS: supervision and manuscript revision. All authors have read and agreed to the published version of the manuscript.

## Funding

This work is the publication of a Master’s thesis of the Master’s program in Clinical Research provided by Dresden International University, Dresden, Germany. The Article Processing Charges (APC) were funded by the joint publication funds of the TU Dresden, including Carl Gustav Carus Faculty of Medicine, and the SLUB Dresden as well as the Open Access Publication Funding of the DFG.

## Conflict of interest

The authors declare that the research was conducted in the absence of any commercial or financial relationships that could be construed as a potential conflict of interest.

## Publisher’s note

All claims expressed in this article are solely those of the authors and do not necessarily represent those of their affiliated organizations, or those of the publisher, the editors and the reviewers. Any product that may be evaluated in this article, or claim that may be made by its manufacturer, is not guaranteed or endorsed by the publisher.
