# Supplementary material for: Intravenous thrombolysis and endovascular therapy for acute ischemic stroke in COVID-19: a systematic review and meta-analysis
Source: Front Neurol. 2023 Aug 15;14:1239953. doi: 10.3389/fneur.2023.1239953 (PMC10482345; doi:10.3389/fneur.2023.1239953)

**Supplementary Material**

1. **Search Strategy**

1.1 MEDLINE

| Query | Sort By | Filters | Search Details |
| --- | --- | --- | --- |
| ("stroke" OR "cerebrovascular disease" OR "ischemic stroke" OR "ischaemic stroke" OR "brain ischemia" OR "cerebral ischemia" OR "embolic stroke" OR "cerebrovascular disorders") AND ("coronavirus" OR "COVID" OR "COVID-19" OR "2019-nCoV" OR "severe acute respiratory syndrome coronavirus 2" OR "SARS-CoV-2") |  | Case Reports, Clinical Study, Clinical Trial, Clinical Trial, Phase I, Clinical Trial, Phase II, Clinical Trial, Phase III, Clinical Trial, Phase IV, Comparative Study, Controlled Clinical Trial, Meta-Analysis, Observational Study, Randomized Controlled Trial, Review, Systematic Review, Adult: 19+ years | (("stroke"[All Fields] OR "cerebrovascular disease"[All Fields] OR "ischemic stroke"[All Fields] OR "ischaemic stroke"[All Fields] OR "brain ischemia"[All Fields] OR "cerebral ischemia"[All Fields] OR "embolic stroke"[All Fields] OR "cerebrovascular disorders"[All Fields]) AND ("coronavirus"[All Fields] OR "COVID"[All Fields] OR "COVID-19"[All Fields] OR "2019-nCoV"[All Fields] OR "severe acute respiratory syndrome coronavirus 2"[All Fields] OR "SARS-CoV-2"[All Fields])) AND ((casereports[Filter] OR clinicalstudy[Filter] OR clinicaltrial[Filter] OR clinicaltrialphasei[Filter] OR clinicaltrialphaseii[Filter] OR clinicaltrialphaseiii[Filter] OR clinicaltrialphaseiv[Filter] OR comparativestudy[Filter] OR controlledclinicaltrial[Filter] OR meta-analysis[Filter] OR observationalstudy[Filter] OR randomizedcontrolledtrial[Filter] OR review[Filter] OR systematicreview[Filter]) AND (alladult[Filter])) |

1.2 Embase

| No. | Query |
| --- | --- |
| #5 | ('stroke'/mj OR 'cerebrovascular disease'/mj OR 'ischemic stroke'/mj OR 'ischaemic stroke'/mj OR 'brain ischemia'/mj OR 'cerebral ischemia'/mj OR 'embolic stroke'/mj OR 'cerebrovascular disorders'/mj) AND ('coronavirus'/mj OR 'covid'/mj OR 'covid-19'/mj OR '2019-ncov'/mj OR 'severe acute respiratory syndrome coronavirus 2'/mj OR 'sars-cov-2'/mj) AND ([adult]/lim OR [aged]/lim OR [very elderly]/lim) AND [2019-2022]/py |
| #4 | #1 AND ('case control study'/de OR 'case report'/de OR 'case study'/de OR 'cohort analysis'/de OR 'comparative effectiveness'/de OR 'comparative study'/de OR 'controlled study'/de OR 'cross sectional study'/de OR 'medical record review'/de OR 'phase 2 clinical trial'/de OR 'phase 3 clinical trial'/de OR 'prospective study'/de OR 'randomized controlled trial'/de OR 'retrospective study'/de) AND ([adult]/lim OR [aged]/lim OR [very elderly]/lim) AND ('Article'/it OR 'Article in Press'/it OR 'Conference Abstract'/it OR 'Conference Paper'/it OR 'Conference Review'/it OR 'Preprint'/it OR 'Review'/it) |
| #3 | #1 AND ('case control study'/de OR 'case report'/de OR 'case study'/de OR 'cohort analysis'/de OR 'comparative effectiveness'/de OR 'comparative study'/de OR 'controlled study'/de OR 'cross sectional study'/de OR 'medical record review'/de OR 'phase 2 clinical trial'/de OR 'phase 3 clinical trial'/de OR 'prospective study'/de OR 'randomized controlled trial'/de OR 'retrospective study'/de) AND ([adult]/lim OR [aged]/lim OR [very elderly]/lim) |
| #2 | #1 AND ('case control study'/de OR 'case report'/de OR 'case study'/de OR 'cohort analysis'/de OR 'comparative effectiveness'/de OR 'comparative study'/de OR 'controlled study'/de OR 'cross sectional study'/de OR 'medical record review'/de OR 'phase 2 clinical trial'/de OR 'phase 3 clinical trial'/de OR 'prospective study'/de OR 'randomized controlled trial'/de OR 'retrospective study'/de) |
| #1 | ('stroke'/exp OR 'stroke' OR 'cerebrovascular disease'/exp OR 'cerebrovascular disease' OR 'ischemic stroke'/exp OR 'ischemic stroke' OR 'ischaemic stroke'/exp OR 'ischaemic stroke' OR 'brain ischemia'/exp OR 'brain ischemia' OR 'cerebral ischemia'/exp OR 'cerebral ischemia' OR 'embolic stroke'/exp OR 'embolic stroke' OR 'cerebrovascular disorders'/exp OR 'cerebrovascular disorders') AND ('coronavirus'/exp OR 'coronavirus' OR 'covid'/exp OR 'covid' OR 'covid-19'/exp OR 'covid-19' OR '2019-ncov'/exp OR '2019-ncov' OR 'severe acute respiratory syndrome coronavirus 2'/exp OR 'severe acute respiratory syndrome coronavirus 2' OR 'sars-cov-2'/exp OR 'sars-cov-2') AND ([adult]/lim OR [aged]/lim OR [very elderly]/lim) AND [2019-2022]/py |

1.3 Cochrane Library

ID Search

#1 ("stroke" OR "cerebrovascular disease" OR "ischemic stroke" OR "ischaemic stroke" OR "brain

ischemia" OR "cerebral ischemia" OR "embolic stroke" OR "cerebrovascular disorders") AND

("coronavirus" OR "COVID" OR "COVID-19" OR "2019-nCoV" OR "severe acute respiratory

syndrome coronavirus 2" OR "SARS-CoV-2") (Word variations have been searched)

2. **Risk of bias assessment** using the Risk Of Bias In Non-randomized Studies - of Exposure (ROBINS-E) tool. (A) Traffic light plot of the domain-level judgements for each individual result and (B) weighted bar plots of the distribution of risk-of-bias judgements within each bias domain.


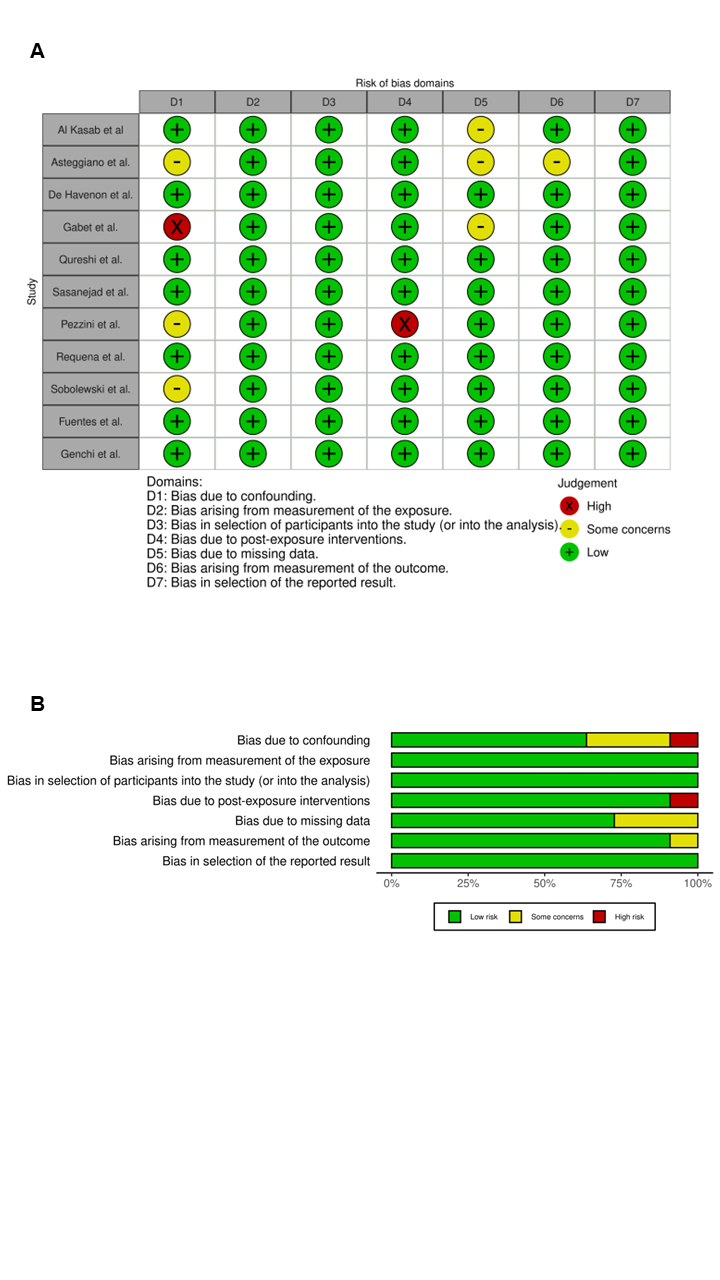


3. **Funnel plot** of all studies providing data on in-hospital mortality


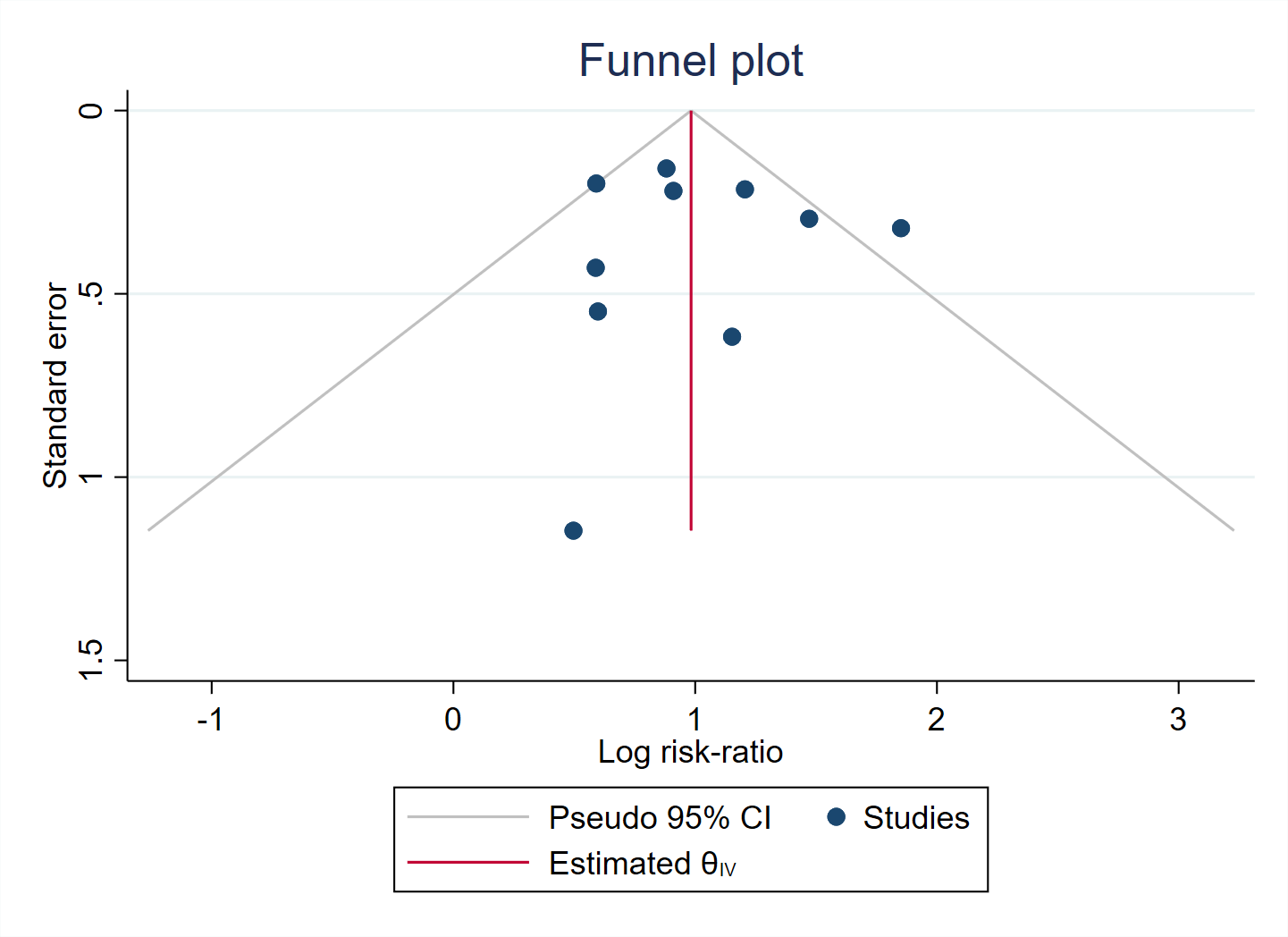

Supplement: Supplementary file 1 [file Data_Sheet_1.docx]
